# Improving the implementation of health workforce policies through governance: a review of case studies

**DOI:** 10.1186/1478-4491-9-10

**Published:** 2011-04-12

**Authors:** Marjolein Dieleman, Daniel MP Shaw, Prisca Zwanikken

**Affiliations:** 1 Royal Tropical Institute, Mauritskade, Amsterdam, the Netherlands; 2 Independent consultant, Geneva, Switzerland

## Abstract

**Introduction:**

Responsible governance is crucial to national development and a catalyst for achieving the Millennium Development Goals. To date, governance seems to have been a neglected issue in the field of human resources for health (HRH), which could be an important reason why HRH policy formulation and implementation is often poor. This article aims to describe how governance issues have influenced HRH policy development and to identify governance strategies that have been used, successfully or not, to improve HRH policy implementation in low- and middle-income countries (LMIC).

**Methods:**

We performed a descriptive literature review of HRH case studies which describe or evaluate a governance-related intervention at country or district level in LMIC. In order to systematically address the term 'governance' a framework was developed and governance aspects were regrouped into four dimensions: 'performance', 'equity and equality', 'partnership and participation' and 'oversight'.

**Results and discussion:**

In total 16 case studies were included in the review and most of the selected studies covered several governance dimensions. The dimension 'performance' covered several elements at the core of governance of HRH, decentralization being particularly prominent. Although improved equity and/or equality was, in a number of interventions, a goal, inclusiveness in policy development and fairness and transparency in policy implementation did often not seem adequate to guarantee the corresponding desirable health workforce scenario. Forms of partnership and participation described in the case studies are numerous and offer different lessons. Strikingly, in none of the articles was 'partnerships' a core focus. A common theme in the dimension of 'oversight' is local-level corruption, affecting, amongst other things, accountability and local-level trust in governance, and its cultural guises. Experiences with accountability mechanisms for HRH policy development and implementation were lacking.

**Conclusion:**

This review shows that the term 'governance' is neither prominent nor frequent in recent HRH literature. It provides initial lessons regarding the influence of governance on HRH policy development and implementation. The review also shows that the evidence base needs to be improved in this field in order to better understand how governance influences HRH policy development and implementation. Tentative lessons are discussed, based on the case studies.

## Introduction

Responsible governance is crucial to national development and a catalyst for achieving the Millennium Development Goals [1]. Poor governance, exemplified by poor accountability and transparency, corruption and limited engagement of communities in health, contributes to ineffective health systems [2]. Since the early 1990s, several institutions have defined governance at state level (United Nations Development Programme (UNDP), the World Bank, Department for International Development (DFID) and International Monetary Fund (IMF), among others) so as to address challenges in development [3]. For health, this term has been operationalized since 2000, by World Health Organisation [4], Pan American Health Organization (PAHO) [5], and Brinkerhoff and Bossert [2], among others. A single definition does not exist, and the definitions used cover similar issues, yet with seemingly different foci. Most notable is that governance in the health sector emphasizes management issues, such as the development of structures for efficient service delivery, as illustrated by PAHO's formulation of essential public health functions [5] and WHO's introduction of 'stewardship' [4]. Less explicit attention seems to be paid to power and interest of stakeholders, in other words, the political aspects of governance.

A definition of governance which includes this political dimension is provided by Brinkerhoff and Bossert [2]: "Governance is about the rules that distribute roles and responsibilities among government, providers and beneficiaries and that shape the interactions among them. Governance encompasses authority, power, and decision making in the institutional arenas of civil society, politics, policy, and public administration".

Whilst governance in health systems has been receiving increased attention [2], to date, governance seems a neglected issue in the field of human resources for health (HRH). This could be an important reason why HRH policy formulation and implementation is often poor. Despite the existence of HRH plans in 45 of the 57 HRH crisis countries [6], in practice HRH policies often do not seem to fit with the local situation, do not respond to health workers' or consumer needs, or are not well implemented [7]. Anecdotal evidence on poor accountability, corruption and limited involvement of communities in HRH policy development and implementation are present. Examples of governance issues in HRH have been described in the literature, such as health workers referring patients to their own private clinic [8], task shifting not being regulated [7], and global health initiatives causing health workers to neglect their tasks for the benefit of the global health initiative (GHI) programs [9].

Apart from poor governance, additional reasons underlying poor HRH policy formulation and implementation include ineffective management strategies and poor management competencies. Management issues have been more often addressed in the HRH literature (e.g. by Fritzen [10] and Buchan [11], among others) but governance is mostly not addressed or not addressed comprehensively. This could in part be due to a more general common tendency to conflate 'management' and 'governance' which are, in fact, very different terms, albeit often closely related. Limited understanding of the relation between success or failure of HRH plans and governance-related issues thus represents an important gap in HRH knowledge, and therefore an opportunity to effectively address poor policy formulation and implementation is missed.

This article describes and analyses published case studies on governance issues impacting on HRH policy implementation at country or district level in low- and middle-income countries (LMIC), with the intention to provide some insights into governance issues in the area of HRH and to put governance more centrally on the HRH agenda. It aims to describe how governance issues have influenced HRH policy development and to identify governance strategies that have been used, successfully or not, to improve HRH policy implementation in LMIC countries. To our knowledge, no such review of human resources for health and governance has yet been undertaken.

## Methods

This is a descriptive literature review, using published case studies which describe or evaluate a governance-related intervention at country or district level in low- and middle income countries (LMIC). We purposely searched and analysed case studies, as we intended to illustrate, with country examples, the positive and negative influences governance can have on HRH policy formulation and implementation, while at the same time keeping the focus on national governance, as opposed to international or clinical (facility level) governance. Although many common aspects appear among different definitions and frameworks for governance in health, these are often described using a variety of terms [12]. In order to address the term 'governance' more systematically, and to allow a simple overview, we use the governance aforementioned definition of Brinkerhof and Bossert [2] as a basis.

In addition, we regrouped the different governance aspects into four dimensions: 'performance', 'equity and equality', 'partnership and participation' and 'oversight' [13], by combining the contents of definitions and frameworks, notably the assessment framework for health systems governance [1], but also including definitions of WHO [4], PAHO[5], World Bank [14] and United Nations Development Programme (UNDP) [15]. An overview of the main elements of these definitions and frameworks is provided in Additional file 1. Each dimension has been defined as follows:

### Performance

Efficiency/effectiveness of HRH policies and plans: demonstrating the political will and commitment to formulate and implement evidence- and needs-based HRH, and to allocate resources (including financial); showing leadership; assuring a high-quality plan and monitoring and evaluation of its implementation

### Equity and equality

Equity and equality in HRH policy formulation, and implementation or inclusiveness of policy; addressing community needs as well as health workers needs.

### Partnerships and participation

Partnerships and participation: being able to effectively work together and having a level-playing field in which groups with different interests and different roles have an opportunity to participate, to bring forward their position and negotiate regarding HRH policies.

### Oversight

Oversight: accountability and rule of law. Accountability is about assuring that those who are responsible for designing and implementing HRH policies are held accountable for their performance. 'Rule of law' refers to, among other things, penalising corruption; addressing fair implementation of and adherence to labour law and civil service regulations on rights and obligations of the workforce; fair implementation of and adherence to accreditation and licensing; regulatory frameworks; and complaints and arbitration mechanisms.

Table 1 provides an overview of how the different components described in articles on governance in the field of HRH were regrouped according to these four dimensions ('performance', 'equity and equality', 'partnership and participation' and 'oversight').

**Table 1:** The four dimensions of governance and corresponding components

Performance	Efficiency and effectiveness, capacity to implement
	Ethics and respect (incl. for citizens)
	Intelligence, information, evidence, m&e
	Policy objectives vs. Organizational structure capacity to implement, decentralization
	Strategic vision, leadership, direction, decision-making process
**Equity and equality**	Fairness, equity, inclusiveness responsiveness

**Partnerships and participation**	Consensus orientation, coalition, partnership
	Legitimacy, voice, participation

**Oversight**	Accountability
	Regulation
	Rule of law, enforcement (incl. corruption control)
	Transparency

### Search strategy

Published case studies were searched for using the following criteria: articles published in English and in peer reviewed journals published from 2006 to January 2010. We used the year 2006 as a starting point because it was the year the World Health Report on the health work force crisis was published. We assumed that this would have been a starting point for (more) attention in the literature on HRH issues, including HRH and governance.

We included case studies of:

- interventions at LMIC country-level or district level aimed at improving and/or analysing governance aspects of HRH; and/or

- assessment of the effects of global governance on the country-level HRH situation.

We excluded articles not published in English, articles on generic HRH assessments, situational analyses of HRH and articles on clinical governance, as literature on clinical governance is mostly focused on facility-level interventions.

We combined various synonymic terms for 'human resources for health' and terms related to governance as determined by the aforementioned major governance frameworks and evaluations published in recent years [1,4,5,14,15]. We specifically searched for studies which used a case study approach. This resulted in the following key search terms:

### Generic terms for human resources for health

Health human resources, health personnel, health staff, health workers, health workforce, HRH, human resources in health, human resources for health.

### Terms related to governance

Accountability, accountable, accreditation, administration, professional associations, civil society, corruption, decentralization, decentralized, decentralize, governance, government, leadership, legislation, licensing, policy analysis, policy implementation, political economy, regulation, stewardship, transparency.

### Databases consulted

In searching for the country/district-level case studies, we consulted Scopus, PubMed and Embase: three databases which include a vast amount of journals that cover health systems, HRH and governance in LMIC.

### Data processing and analysis

The authors of this article constituted the research team, and were assisted by a librarian to search for abstracts. The initial search for articles was done by the librarian; all abstracts were screened by two researchers. Full text of the articles that met the inclusion criteria were read and analysed by two researchers, independently from each other. For data analysis we developed an analytical framework that was tested by jointly analysing one article. The analytical framework included a description of the context, the intervention and results and a description on governance dimensions, based on the governance dimensions presented in this article. Each article was discussed by the two researchers and when no consensus was reached, the third researcher was asked to read and analyse the article.

## Results

**Figure 1:**
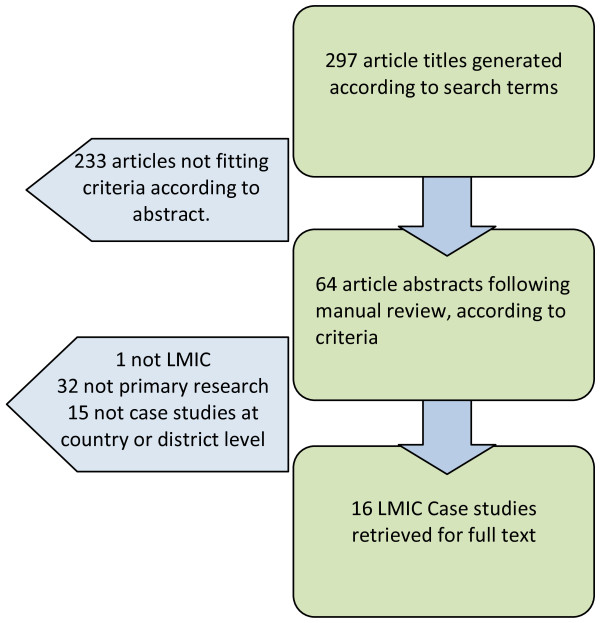
**Literature search: selection of primary studies on HRH governance in LMIC**.

In total, sixteen case studies were included in the review and Figure 1 summarizes the search results.

Additional file 2 gives an overview of these studies. Most of the case studies relating to HRH and governance in English come from Africa (6) and Asia (8). While the lower proportion from Latin America (2) and the transitional economies in Europe (0) could be explained by our literature search concentrating on papers written in English, the same is probably not the case for the lack of studies from Australasia/South Pacific. In the English language, at least, there is a serious gap in the literature from such countries. When looking at the affiliation of the authors, the overwhelming majority of lead authors of the sixteen selected case studies were from northern institutes, with only three being written by lead authors who were members of national research institutions of the country concerned (Liu, China [16]; George [17], India; and Burns [18], South Africa). Another seven were written by international organizations, non-governmental organizations (NGO) and international donors. A similar number (6) represented authors from foreign research institutions.

The following section reports on the results of the literature review regarding the influence of the different dimensions of governance on the respective HRH situation or on HRH policy development. As most of the selected studies covered several governance dimensions, articles are cited under several governance dimensions so as to illustrate and give examples.

### Performance

#### Decision making

No case studies describe how decision making for HRH policy development takes place. A number of cases show that a lack of participation in decision making can hamper successful implementation, for instance unions, did not participate in designing hospital reforms--including reforms in HRH policies for hospital workers --in Costa Rica. This might have contributed to their resistance to change [19].

#### Evidence-based policy formulation

Two case studies discuss intelligence, information and/or evidence pertaining to HRH [20,21]. The case study on decentralization (and also recentralization) of HRH responsibilities in Indonesia found that monitoring of stocks and flows of health workers worsened, and that HRH information suffered, following decentralization [20]. In Laos, aid effectiveness efforts included new structures to share analysis of staffing quota systems--resulting in a joint HRH situational analysis--and arrangements for the government to develop a new HRM database, supported by UNICEF [21].

#### Strategic vision, leadership and direction

Eleven case studies addressed leadership, vision and strategic direction [9,16-18,21-27].

Examples of the importance of leadership and having a vision are provided from a variety of situations: post-apartheid government vision of a fairer South Africa was behind the motivations for the development and implementation of the Mental Health Care Act 2002 [18]; and the bold leadership of two major stakeholders was enough to foster major change in direction and donor collaboration, including resource allocation, in Malawi [25]. In Botswana, presidential-level commitment greatly facilitated the creation of the public-private mechanisms to increase access to HRH in HIV/AIDS[23]. In Afghanistan, a similar endorsement of the initiative to develop a new accreditation system for midwifery education by the government and Ministry of Public Health--by ceding regulatory authority to the accreditation board--most likely helped in expediting the programme [26]. In Nepal, the politics of conflict resulted in the decision by the leadership to ban health workers from treating rebels (referred to as 'terrorists'), which caused 20 000 people to forfeit health care [22]; and the Zambian case study on HRH implications of Global Health Initiatives (GHI) demonstrates how leadership in HRH policy is compromised by the resultant dependency on external actors [9]. Teela et al. [27], 2009 show that, in Myanmar, community leadership was created in maternal health care. The program also created a sense of leadership among the health workers themselves, who felt they were more than 'just' a health worker, but also leading figures in their communities.

Munga et al. describe decentralization of HRH recruitment, which created an opportunity for an HRH planning that was more responsive to local needs. However, a major stumbling block was that the lack of power of district level authorities restricted them from exercising leadership in their management of HR and in disputes with an over-controlling central authority [24]

#### Reforms and decentralization

Five articles had decentralisation as a primary focus [16,18,19,24,28]. Other articles also provided lessons for decentralisation.

In post-conflict Guatemala, moving services and responsibilities closer to the communities was seen by local people as the government showing interest in their needs. This enhanced trust in the government and its services and was followed by signs of improved health indicators and immunisation coverage in the corresponding communities [28]. In Tanzania, it was found that decentralisation increased flexibility in planning and ownership of local services and this is likely to have increased retention of health workers [24].

Despite the intention, among other objectives, to improve HRH management through decentralization, there were several experiences of decentralization that impacted negatively on the health workforce. For example, decentralization of the health system and creation of new policies that integrate increased responsibilities for care at the primary level may increase the workload of local-level health workers, especially when not coupled with a revised staffing plan at that level, as shown in Costa Rica, Guatemala and South Africa [18,19,28]. Typically, in such cases the administrative burden is also augmented, leaving less time and human resources for actual care or treatment work [18]. Studies from China and South Africa point out under-preparation of staff, managers and administrators at the decentralized level and claim that many health workers received little or no communication from the central authorities on the nature of the new policy and how it was to be dealt with at a local level [16,18]. In China, decentralization of the health sector to improve HRH management resulted in a distinct risk of nepotism at the lower level [16].

The lack of clarity that often arises in implementation of decentralization creates mismatches such as transfer of roles and responsibilities without a similar transfer of adequate resources. Accountability becomes unclear and transparency can be lacking, with problems of patronage occurring at decentralized levels as well.

### Equity and equality

Equity and equality was addressed in five articles, albeit not directly as a core focus of the studies [16,17,20,24,28].

Maupin [28] reports that outsourcing of care provision to NGOs at the local level in Guatemala showed there were early indications that equity improved, although the planning had not adequately taken into account local HRH realities and perceptions, thus not optimising the opportunities to improve access to care by including local non-governmental organization (NGOs) in service provision.

Three case studies demonstrate the relationship between HRH and equity and equality in access to care through decentralization: those from Indonesia, China and Tanzania [16,20,24]. In Tanzania, it was found that decentralized recruitment can provide a planning process that is more responsive to local health service needs, contributing to reducing inequalities and inequities in service provision. However, increased bureaucracy in practice and numerous conflicts between local and central authorities--with the autonomy of the former often being over-ridden by the latter in recruitment procedures--have resulted in the chances of successful recruitment, distribution and retention of health workers being compromised. Decentralization actually exacerbated distribution imbalances between areas in Tanzania rather than improving them [24].

In China, local managers were not prepared to deal with management of human resources in the health sector [16]. This resulted in inappropriate human resource (HR) management at local level, causing decentralization to negatively impact on service provision, including inequity of service provision. Furthermore, the depth and nature of decentralization was unclear, and there were financial and managerial tensions related to incentive systems and corruption/nepotism.

Heywood et al. demonstrate that in Indonesia, the HRH situation has moved from centralization and compulsory service in disadvantaged areas, to decentralization with districts being more involved in HRH management, to re centralization of contracts. In the process, the removal of compulsory service in disadvantaged areas--and a lack of clarity and information on private practice--have led to many graduates moving to the private sector, without having to register. They are thus lost to the system, many setting up clinics in urban areas. As a result, there are fears that quality and use of health services by the most disadvantaged people will suffer, exacerbating existing inequities [20].

One case study touches on the dimension of equity and equality at health worker level. George [17] reports on perspectives of government health administrators and health workers in the district of Koppal, India, on accountability mechanisms within the health department. Health workers and administrators have to deal with corruption, favouritism, nepotism and bias resulting from informal management systems. The study shows how local context in terms of traditions and culture can raise challenges in dealing with inequity and equality among health workers [17].

### Partnership and participation

In the 16 selected articles, partnership and participation is rarely the primary focus and yet it plays an important secondary role in most [9,17-23,25-29]. The types of partnership described in the case studies and whether they contribute positively--or negatively--to improved health and HRH outcomes is explored below.

### Partnerships between governments and development partners

Three case studies describe how HRH policy development is influenced by the relationship between governments and development partners/funding agencies. Two report on positive experiences with donor-government coordination [21,25]. Dodd [21] describes how, in Laos, harmonization of donors' and governments' priorities led to more coherent support from donors, which in turn provided an incentive to governments to develop HRH policies that donors could support. Donor coordination in Malawi was possible because of the commitment of two lead donors (DFID and United States Agency for International Development (USAID)) [25]. A lead donor was necessary to convince other donors to pay salary top ups, because donors had for so long signalled that they could not help address pay.

A more negative experience was described in a case study in Zambia [9], where a global health initiative (GHI) funded extra activities to increase access to AIDS treatment, without budgeting for more staff. This resulted in a significant increase in workload of health workers and administrators, since there was a lack of new staff brought in. Furthermore, staff members were recruited from their public service positions into the GHI organizations themselves. In terms of partnership, the relationship between the national system and donors became one of dependency.

### Partnership and participation in fragile states

Three case studies highlight participation in conflict areas [22,27,29]. Civil war, by definition, segregates, and this is exacerbated in examples such as the government banning of treating Maoist rebel forces, which resulted in deterring tens of thousands of Nepalese from seeking medical treatment [22]. One of the main recommendations concerns proposals for partnership *following *the conflict: retraining and mobilizing Maoist health workers following the conflict would not only help to boost health coverage, but would serve as an olive branch for conflict transformation and peace building, bringing both sides together [22]. Teela et al. [27] present a picture where participation, voice and legitimacy were key to a programme's development and success in conflict situations, as formal care provision was often not functioning. By creating an environment of mutual trust between the communities and other actors involved (often development partners and/or funding agencies) success was feasible, despite the wider instability in the region. A community-based approach created a sense of community ownership and inclusiveness. In terms of a participative process, community meetings and communication were considered vital prior to implementation. Such meetings were also an opportunity for stakeholders to engage with the population and demonstrate their competence, equally fundamental to achieving community trust and promoting increased access [27]. Lee et al. [29] describe how community partnership with a local ethnic health department demonstrated that village health workers are capable of successfully implementing malaria control interventions among internally displaced persons in a diverse, community-run team.

### Partnerships with the private sector

The health workforce available to provide services can be increased by engaging the private sector. This was described in two case studies. Dreesch et al. [23] showed that in Botswana comprehensive partnerships across the board greatly improved the effectiveness of service delivery. Partnerships with the private sector, and the mechanisms that allow it, were key, maximizing use of the available human resources for health in the country for the treatment of and attention to HIV/AIDS. In Tanzania, there is a potential of a similar private sector partnership in contributing to the MDG target of increasing skilled attendance at delivery by allowing 'retired' midwifery workforce in Tanzania to open private practices in rural areas [30].

In Guatemala, outsourcing to local NGO's did not always work out, as many of the commissioned NGOs were soon acting as administrators of care rather than direct implementers. Furthermore, some of the most qualified NGOs decided not to take on the outsourced role in the interest of retaining their autonomy, and other NGOs with no or little experience in delivering health care took up the contract resulting in inefficient parallel systems of care [28].

### Participation of health worker associations and unions

In Costa Rica the impact of hospital management reforms on absenteeism, the sick-leave policy and the design of management contracts was not as positive as expected, and the authors mention that the current management reforms met union resistance. They hint that this may also have been a reason for their relative failure upon implementation. The authors emphasize the importance of involving and reaching an agreement with the unions first [19].

### Oversight

Six case studies discussed matters relating to the governance dimension of 'Oversight'. A common theme and concern within the literature regards the challenges of political interference at the local level, related to implementation of decentralization, internal accountability mechanisms, aid effectiveness and service delivery in conflict settings [16,17,21,22,24]. In none of the studies were interventions to deal with these interferences discussed.

Four case studies described matters relating to regulation [21,24,27,29]. Dodd et al. [21] showed that in Laos, efforts to improve aid effectiveness for HRH led to improved accountability both from a point of view of the donor and that of the government. However, there was a certain amount of resistance in the form of a lack of commitment from certain civil service administrators, for whom the proposed new system would result in personal loss. Despite this resistance, the aid effectiveness agenda improved governance for HRH and it was furthermore used as a starting platform for reformed workforce planning, regulation and financial management.

The study in Koppal district in India describes how corruption facilitates the circumvention of accountability systems[17]. It describes how supervision and disciplinary action are rarely implemented in a straightforward manner in this particular district, and incentives to follow the rules (or actions) that were agreed upon are weaker than personal incentives. In this case, accountability is found by the authors to be best characterized as a nuanced social process, where power relations are negotiated by multiple actors with both positive and negative effects for HRH. Informal relations can distort regulatory systems, and in local settings where there is a tendency for corruption, they can even be described as sustaining the (local) health system [16,17]. Accountability is about having the right checks and balances put into place [16]. Dodd et al. postulate that if financial regulations were made more flexible at the local level, health managers at that level would be then more empowered to innovate tailor-made incentives to attract health workers [21].

### Oversight during conflict

Two cases addressed oversight in conflict-affected eastern Myanmar [27,29]. These cases showed that when a population is isolated, cut off, displaced or neglected, a community oversight mechanism can be established and can function if a seed pool of resources is present (i.e. a critical initial number of educated staff). Regulatory mechanisms evolved in parallel with inbuilt monitoring and evaluation feedback loops. Thus, as successes and failures became more apparent, adjustments in training and delegated responsibilities to community health workers and maternal health workers were adapted in a continuing quest for improved care and expanded access. Devkota et al. [22] showed that in Nepal, during a full-blown conflict, political interference, instability, favouritism and other concerns--i.e. a lack of unified rule of law--resulted in health workers being siezed by rebels, medicines and equipment being stolen and false reports being made.

## Discussion

This review shows that the term 'governance' is neither prominent nor frequent in recent HRH literature, and that governance aspects deserve specific attention in HRH policy formulation and implementation. In this article, we have attempted to address a new area for the HRH field in a comprehensive way, and to show that a lot of work--in terms of conceptualization, evidence building and documentation of successful strategies to improve governance--still needs to be done. The selected case studies are dedicated to aspects related to or falling under the concept of governance; not however, to governance and HRH as a whole. Moreover, while there is much to say about each case, drawing conclusions on how each element of governance effects HRH policy development is not possible, due a lack of evidence. Despite these limitations, the group of case studies as a whole allow us to conclude that there are clear indications that governance issues have an impact on HRH policy development and implementation, and on HRH performance, contributing to efficiency and effectiveness of health services delivered by health personnel.

The case studies allow us to draw a number of lessons, these are presented below.

### Performance

The governance dimension of performance covers several elements that could be considered at the origin and at the core of governance of HRH, e.g. efficiency, effectiveness, ethics, vision, leadership, information, evidence and capacity to implement, with decentralization being a particularly prominent issue. However, in the case studies, the decision-making processes are most of the time not clearly described. A lack of insight on how decision-making takes place and who is involved hampers understanding of the reasons why certain HRH policies are selected (and others not); and why certain policies are successfully implemented (and others not). Political economy studies can provide useful insights, but these are uncommon in the field of HRH. Moreover, the case studies rarely explain what (if any) evidence was used to develop plans and to formulate policies, and how financial resources were mobilized and allocated. This is extremely important, as a recent review of HRH policies showed that although 71% of the 45 existing HRH plans included a budget for implementation, only 42% had mentioned appropriate investment of the national government or plans to increase investment for implementation of HRH plans [6].

The case studies demonstrate efforts to expand and diversify the current HRH base using creative structures and innovative approaches. Examples of new approaches to expand the HRH base are developing new cadres such as context-specific community level cadres or new lower cadres or redeploying retired health workers, such as midwives. Other approaches to expanding the HRH base are private sector integration, contracting/outsourcing to NGOs, or--in post-conflict situations--incorporating former rebels. In examining these and other potential solutions more closely, we should simultaneously determine what governance aspects are important for these innovations to most successfully become part of the system. The case studies show the importance of leadership in successful governance, and this includes the careful and clear delegation and devolution of leadership during decentralization or other health sector reforms.

### Equity and equality

The articles show that although improved equity and/or equality was, in a number of interventions, a goal (mostly as an eventual objective or implicit in the values underlying policies and in the language used to articulate them), inclusiveness in policy development, and fairness and transparency in policy implementation, were often not adequate to guarantee the corresponding desired health workforce scenario (i.e. one that expresses and embodies the values of equity and equality to the extent intended prior to implementation). In several cases, a lack of clarity in roles and responsibilities between different levels, or in preparation of decentralization of functions, hampered the attainment of increased equity. Other reasons for failure were cumbersome bureaucracy, loss of staff to other sectors, the blurring of lines between informal and professional relations, the inadequacy of NGO adoption of certain public responsibilities, and corruption. Although it could be argued that matters pertaining to equity and equality lie behind much of governance and its intentions, in the case studies we reviewed it seems rarely explicitly aimed for in policies, nor discussed in the articles.

### Participation and partnerships

Forms of partnership and participation described in the case studies are numerous and offer different lessons. Partnerships and participation are important for assuring broad ownership of HRH policies and plans, and they are addressed in all articles. What is striking, though, is that in none of the articles was partnership the core focus; and also that no examples were identified regarding community partnerships in HRH policy development, nor implementation in stable states.

Overall, there appears to have been a shift in the way in which the decision to partner and collaborate with other actors is taken. More traditionally, it is the government that is looked to, to set up governance structures. However, with the advent of NGOs and a new aid architecture, more power and leadership is shifted to other partners, and this influences the types of partnerships, their composition and their own respective policies. Partnerships with development partners through harmonization and aid effectiveness efforts can lay new ground and trust for boosting efficiency and performance, and they can also stimulate improved collaboration between government sectors. On the other hand, programs separately funded by global health initiatives (GHI) may enhance treatment capacity in the short term, but one case study showed that there might be a risk of unsustainability and dependency upon GHI funds. Partnership with the private sector seems to hold promise for maximizing staff availability and access to care by creating innovative service delivery methods, and it would be useful to have more learning on this, also from other sectors. Additionally, it is recommended to include unions, from inception, in plans to reform policy, so as to avoid resistance from professional groups during implementation.

In the case studies describing fragile states, community partnerships and involvement in policy design and implementation appear especially important, where agreement at the community level seems to create a solid basis for bottom-up state recovery. A lesson from these articles was that gaining the trust of the community and health workers involved is key to supply meeting demand [22,26-29]. The cases also show that immediately after conflict, there are opportunities for real change in governance and systems.

### Oversight

Six case studies provide experiences with aspects included in the dimension 'oversight'. At the same time, this overview demonstrates the dearth of information that has been published under the dimension 'oversight', and particularly the lessons learned. A common theme in the HRH literature falling under the domain of oversight is that of local-level corruption, affecting, amongst other things, accountability and local-level trust in governance, and its cultural guises. It is commonly cited that as one approaches the local level, the separation between professional, informal, cultural and corrupt practices and contexts becomes blurred. Experiences with accountability mechanisms for HRH policy development and implementation were lacking in the case studies as well, and more documentation is required on this area. Another domain for which no case study was identified regarding the oversight dimension is the domain of regulation, in particular regulation of the profession. The role of professional councils is important in this area, and deserves (more) attention in research, and in documentation of their experiences in regulating health cadres.

### Use of framework

The framework that was used to describe and group different aspects of governance was a useful start to assist in drawing common lessons across the case studies for each dimension. The framework assisted in disentangling the broad concept of governance and helped in identifying what governance dimensions are addressed and to what extent. For instance, by regrouping the case studies according to the different dimensions, it became clear that little explicit attention was paid to accountability and to equity and equality. At the same time, regrouping demonstrated how broad and complex the term 'governance' really is. Perhaps unwittingly, most articles do not explicitly define the terms that they use to address the various governance dimensions.

Whilst this allows us to use various examples from the same article to illustrate observations on different dimensions of governance, at the same time it was sometimes difficult to judge which governance dimension within a particular intervention or situation had had the most significant effect or was the most important aspect. It also proved difficult to avoid repetition, as an example could be interpreted in different ways, e.g. covering partnership, but also covering accountability (e.g. Dodd et al. [21] or Devkota et al. [22]).

Overall, decentralization seems to dominate the literature on HRH and governance. In the framework, we placed it under the dimension of 'performance', but in reality it cuts across and includes partnerships, oversight and equity/equality. The dimension of 'equity and equality' is another dimension that could be debated, as it can also be seen as a result of improved partnerships, performance and oversight. This framework would need to be further tested so as to allow adaptation and refinement, and to allow for drawing lessons across interventions. At the same time, this paper is a plea to authors to make explicit and to define governance concepts that are used in HRH interventions and studies, and to develop a common governance vocabulary.

## Conclusion

This review provides initial lessons regarding the influence of governance on HRH policy development and implementation. It also shows that more information is required to assist in improving the evidence base in this field, therefore increasing the understanding of how the different governance dimensions influence HRH policy development and implementation. In fact, governance to improve HRH must be viewed as inseparable from the wider health system and state governance within which it is integrated. It is likely that, at country level, important lessons can be drawn from experiences with the different governance dimensions at health system or state level.

As expressed in the respective sections above, this review also shows the need to increase research on the influence of the four governance dimensions on HRH, as a number of questions remain to be answered. From the results presented in this article, further research questions could be formulated. Examples, by dimension, are:

### Performance

• How does decision making in HRH take place?

• How can political interference be dealt with?

• What experience from other countries can be used as a basis for intervention development in expanding the human resource base?

### Equity/equality

• Are needs of vulnerable groups taken into consideration when HRH strategies are formulated?

• What are mechanisms to improve equity and equality among health workers?

### Partnerships and participation

• Which partnerships and what level of participation are important to influence change?

• How have partnerships influenced HRH policy making and implementation?

### Oversight

• What experiences exist regarding accountability mechanisms at national level, at community and at district level?

• How can regulation facilitate access to services?

These questions will have context-specific answers, and therefore case studies at country level could go a long way in clarifying how the concept of governance for HRH has been operationalized in different contexts and what efforts have been put in place for improvement.

## Competing interests

The authors declare that they have no competing interests.

## Authors' contributions

MD, DS and PZ formulated the search strategy and selected, read and analysed articles. DS drafted the report on which the article is based. MD and PZ reviewed the report. MD drafted the article. DS and PZ provided feedback. All authors read and approved the final manuscript.

## Supplementary Material

Additional file 1 **Governance: overview of main elements of definitions and frameworks**. Click here for file

Additional file 2 **Selected case studies**. Click here for file
