# Supplementary figures and images for: Improving the implementation of health workforce policies through governance: a review of case studies
Source: Hum Resour Health. 2011 Apr 12;9:10. doi: 10.1186/1478-4491-9-10 (PMC3094272; doi:10.1186/1478-4491-9-10)

##
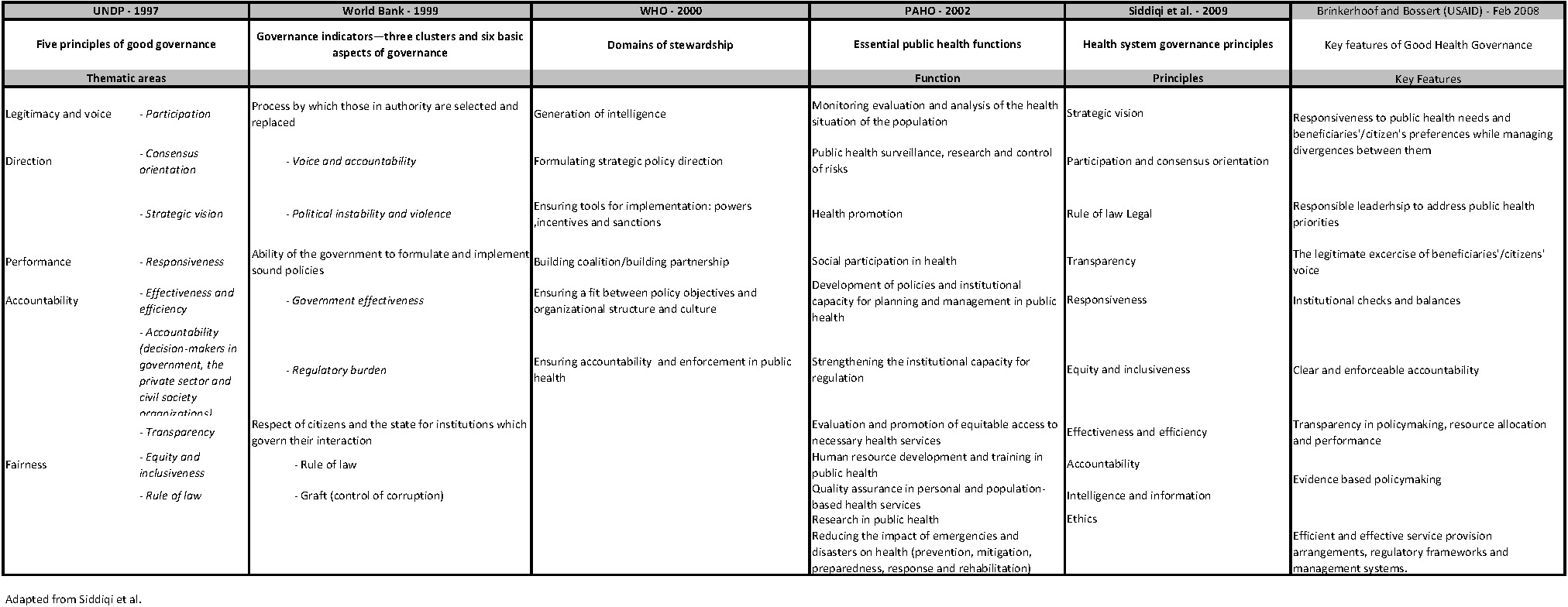
 Additional file 1 - Governance: overview of main elements of definitions and frameworks

Supplement: Additional file 1 — Governance: overview of main elements of definitions and frameworks. [file 1478-4491-9-10-S1.DOC]
