# Supplementary material for: Improving the implementation of health workforce policies through governance: a review of case studies
Source: Hum Resour Health. 2011 Apr 12;9:10. doi: 10.1186/1478-4491-9-10 (PMC3094272; doi:10.1186/1478-4491-9-10)
Supplement: Additional file 2 — Selected case studies. [file 1478-4491-9-10-S2.DOC]

# Additional file 2 - Selected case studies

| **Full reference** | **Key issues addressed** |
| --- | --- |
|
| **Burns JK. Implementation of the Mental Health Care Act (2002) at district hospitals in South Africa: Translating principles into practice. South African Medical Journal 2008;98(1):46-49.** | Outlines rationale for MHCA2002, discuss the problems encountered and offer solutions to the problem of 'translating principles of mental health into practice' |
| **Devkota B, van Teijlingen E.: Case study of rebel health services in Nepal, Asia Pac J Public Health 2009; 21;377 DOI: 10.1177/1010539509342434** | Systematically reviews the health consequences of Nepal's armed conflict waged by Maoists and development and trajectory of their health workers. |
| **Dodd R, Hill PS, Shuey D, Anutnes AF (2009) Paris on the Mekong: using aid effectiveness agenda to support human resources for health n the Lao People's Democratic Republic, Human Resources for Health 7:16** | Examines the potential of aid effectiveness to positively influence human resources for health |
| **Dreesch N, Nyoni J, Mokopakgosi O, Seipone K, Kalilani JA, Kaluwa A and Musowe V (2007) Public-private options for expanding access to human resources for HIV/AIDS in Botswana, Human Resources for Health, 5:25** | Describes the involvement of the private sector to provide HIV/AIDS services |
| **García-Prado A, Chawla M. The impact of hospital management reforms on absenteeism in Costa Rica. Health Policy Plan. 2006;21(2):91-100** | Tests whether the introduction of new organizational arrangements was followed by lower absence rates than those registered previously. |
| **George A (2009) "By papers and pens, you can only do so much": views about accountability and human resource management from Indian government health administrators and workers, International journal of health planning and management 24(3): 205-224** | Details how accountability functions internally within the health department from the perspective of government health administrators and health workers. It examines how two key human resource management functions concerned with internal accountability, supervision and disciplinary action, are implemented in Koppal's government primary health care services |
| **Hanefeld J and Musheke M (2009) What impact do Global Health Initiatives have on human resources for antiretroviral treatment roll-out? A qualitative policy analysis of implementation processes in Zambia, Human Resources for Health, 7:8** | Examines the impact of GHIs on human resources for ART roll out in Zambia, national, in one province and 2 districts. The focus is on GHI's ability to retain and retrain staff and on unintended consequences of their programmes on HRH |
| **Heywood P, Harahap N Human Resources for health at the district level in Indonesia: the smoke and mirrors of decentralization Human Resources for Health 2009,7:6 doi: 10.1186/1478-4491-7-6** | Determines stock of human resources for health in 15 districts, their service status and primary place of work. Assessment of effect of decentralization on management of human resources and implications for future |
| **Lee CI, Smith LS, Shwe Oo EK, Scharschmidt BC, Whichard E, Kler T, et al. Internally displaced human resources for health: Villager health worker partnerships to scale up a malaria control programme in active conflict areas of eastern Burma. Global Public Health 2009 2009/;4(3):229-241.** | Describes how internally displaced villagers facilitated rapid expansion of the programme: a local ethnic health department demonstrated that village health workers are capable of implementing malaria control interventions along internally displaced persons (IDPs) |
| **Liu X, Matrineau T, Chen L, Zhan S, Tang S Does decentralization improve human resource management in the heath sector? A case study from China Social Science and Medicine 63 (2006) 1836-1845** | Examines whether decentralization resulted in improved management of human resources in the health sector |
| **Maupin JN. 'Fruit of the accords': healthcare reform and civil participation in Highland Guatemala. Soc.Sci.Med. 2009 Apr;68(8):1456-1463.** | Examines the contracting process in the government contracting of NGOs in Guatemala (a central strategy of neoliberal reforms in Guatemala and Central America); to address issues of equity, efficiency and quality of services as well as process of decentralization. |
| **Munga MA, Songstad NG, Blystad A and Maestad O (2009) The decentralization-centralization dilemma: recruitment and distribution of health workers in remote districts of Tanzania, BMC International Health and Human Rights, 9:9** | Highlights the experiences and challenges associated with decentralization and the partial re-centralization in relation to the recruitment and distribution of health workers |
| **Palmer D (2006) Tackling Malawi's Human Resources Crisis, Reproductive Health Matters 14:27): 27-39** | Describes how donors support the Malawian government in addressing the HRH crisis |
| **Rolfe B, Leshabari S, Rute F, Murray S The crisis in human resources for health care and the potential of a 'retired' workforce: case study of the independent midwifery sector in Tanzania Health Policy and Planning 2008: 23; 137-149** | Presents drivers and inhibitors acting upon the development of a one new element of non-government provision in Tanzania- the small-scale independent midwifery practice and consider what contribution this sector may be expected to make to the MDG target of increasing skilled attendance at delivery |
| **Smith JM, Currie, S, Azfar P, Rahmanzai AJ (2008) Establishment of an accreditation system for midwifery education in Afghanistan: maintaining quality during national expansion Public Health 122, 558-567** | Presents experience of design and implementation of a simple structured system for accrediting midwifery education programmes in Afghanistan.  Relates process for guiding an educational system through rapid expansion in post-conflict environment |
| **Teela KC, Mullany LC, Lee CI, Poh E, Paw P, Masenior N, et al. Community-based delivery of maternal care in conflict-affected areas of eastern Burma: Perspectives from lay maternal health workers. Social Science and Medicine 2009;68(7):1332-1340.** | Investigates perceptions of community-based workers providing essential maternal and newborn health services directly to vulnerable communities in a conflict setting. |
